# Impact of Anti-Inflammatory Agents on the Gene Expression Profile of Stimulated Human Neutrophils: Unraveling Endogenous Resolution Pathways

**DOI:** 10.1371/journal.pone.0004902

**Published:** 2009-03-19

**Authors:** Mireille St-Onge, Aline Dumas, Annick Michaud, Cynthia Laflamme, Andrée-Anne Dussault, Marc Pouliot

**Affiliations:** Centre de Recherche en Rhumatologie et Immunologie du CHUQ and Department of Anatomy-Physiology, Faculty of Medicine, Laval University, Quebec City, Quebec, Canada; Centre de Recherche Public de la Santé (CRP-Santé), Luxembourg

## Abstract

Adenosine, prostaglandin E_2_, or increased intracellular cyclic AMP concentration each elicit potent anti-inflammatory events in human neutrophils by inhibiting functions such as phagocytosis, superoxide production, adhesion and cytokine release. However, the endogenous molecular pathways mediating these actions are poorly understood. In the present study, we examined their impact on the gene expression profile of stimulated neutrophils. Purified blood neutrophils from healthy donors were stimulated with a cocktail of inflammatory agonists in the presence of at least one of the following anti-inflammatory agents: adenosine A_2A_ receptor agonist CGS 21680, prostaglandin E_2_, cyclic-AMP-elevating compounds forskolin and RO 20-1724. Total RNA was analyzed using gene chips and real-time PCR. Genes encoding transcription factors, enzymes and regulatory proteins, as well as secreted cytokines/chemokines showed differential expression. We identified 15 genes for which the anti-inflammatory agents altered mRNA levels. The agents affected the expression profile in remarkably similar fashion, suggesting a central mechanism limiting cell activation. We have identified a set of genes that may be part of important resolution pathways that interfere with cell activation. Identification of these pathways will improve understanding of the capacity of tissues to terminate inflammatory responses and contribute to the development of therapeutic strategies based on endogenous resolution.

## Introduction

Neutrophils constitute the majority of circulating leukocytes and are often the first cells to migrate toward inflammatory lesions, where they exert host defense functions including the phagocytosis of cell debris and invading microorganisms, the generation of oxygen-derived reactive agents and the release of proteolytic enzymes [1]. In response to specific stimuli, neutrophils can synthesize and release an array of factors such as anti-microbial proteins and extracellular matrix proteins as well as several cytokines and chemokines and thereby play a major role in orchestrating early stages of the inflammatory response [2]. Although recurrent infections in patients with defective neutrophil function confirm their importance in host defense, these cells also bear enormous destructive capacity and can elicit significant tissue damage. Unchecked activation of neutrophils is associated with pathological states such as ischemia, sepsis, chronic obstructive pulmonary disease and rheumatoid arthritis [3]–[5]. It is therefore of both fundamental and clinical interest to gain understanding of not only the mechanisms that promote neutrophil functions, but also of those that can restrict such activation and bring about the resolution of inflammation.

Adenosine, through activation of the A_2A_ receptor (A_2A_R) subtype, ranks among the most potent agents limiting the inflammatory activities of neutrophils. One of the first reports on this matter, published more than two decades ago by Cronstein et al. [6], determined that the autacoid inhibited superoxide production resulting from inflammatory stimuli. Interest in adenosine and its receptors has since fuelled major research efforts, which have contributed to increased appreciation of their pivotal importance in limiting inflammation [7]–[9]. High concentrations of extracellular adenosine can be found in vivo in traumatized tissues and this autacoid may have a role in reducing the accumulation of leukocytes at the site of injury [10]. A paramount role for the A_2A_R subtype in mediating anti-inflammatory activities has been for all practical purposes established in previous studies [11]–[16]. The cyclic-AMP-elevating G_s_-protein-coupled A_2A_R subtype modulates key pro-inflammatory neutrophil functions such as superoxide generation, de-granulation and adhesion (reviewed in [17]). Endogenous adenosine and A_2A_R agonists have shown to be potent inhibitors of leukotriene and platelet-activating factor synthesis [13], [18]–[20] and in contrast, to stimulate COX-2 expression in neutrophils [21], [22], thus increasing the capacity of these cells to produce prostaglandin E_2_. This shift in the profile of lipid mediator production from leukotrienes to prostaglandin E_2_ may contribute to preventing subsequent neutrophil-elicited inflammatory events. Recently, our laboratory reported that A_2A_R activation had a striking inhibitory impact on the in vitro and in vivo generation of tumor necrosis factor α and several other neutrophil-derived cytokines and chemokines [23], confirming a preeminent role for adenosine in restricting neutrophil activation. Most of the anti-inflammatory activities of this autacoid through A_2A_R engagement are thought to involve a rise in intracellular cyclic AMP concentration [22], [24], [25]. Prostaglandin E_2_, acting through its own set of receptors, is also a potent inhibitor of neutrophil inflammatory functions and can, similarly to adenosine, modulate pivotal neutrophil effector functions such as chemotaxis, aggregation, superoxide production, lysozyme release and leukotriene B_4_ production by raising intracellular cyclic AMP concentration above basal levels [22], [26]–[33]. Adenosine and prostaglandin E_2_ thus clearly stand out as two major anti-inflammatory signals, while elevated intracellular cyclic AMP concentration, which can be pharmacologically achieved with a combination of the adenylate cyclase activator forskolin and of the phosphodiesterase IV inhibitor RO-20-1724, often appears to accompany their actions. However, the gene activities that control inflammation resolution pathways remain poorly understood.

In the present study, we used DNA microarray technology and real-time PCR to examine the impact of major anti-inflammatory signals, namely A_2A_R activation, prostaglandin E_2_ and elevated intracellular cyclic AMP, on the gene expression profile of human neutrophils stimulated by known inflammatory agonists. We have identified a group of genes for which mRNA levels were significantly altered by anti-inflammatory signals. This may indicate their involvement in pivotal molecular signaling pathways associated with the resolution of inflammation.

## Results

### Gene expression in stimulated human neutrophils

Microarray data is conform to the MIAME guidelines; unsupervised, raw data was deposited in the GEO database (geo@ncbi.nlm.nih.gov), submission number: GSE14465. Initial analysis of the DNA microarray chips using the Affymetrix software indicated that approximately 15,000 of the 54,675 sequences recognized in the array (i.e. 27.5%) are expressed by resting human neutrophils. Comparison between resting and neutrophils stimulated with a mixture of pro-inflammatory agonists for 30 min, revealed 1,152 differentially expressed sequences, of which 401 corresponded to known proteins [34] and are listed in Supplementary Table S1 in descending order of the expression differential magnitude. Using the Kegg pathway database, we selected genes for further examination, based on their potential implication in immune response processes (http://www.genome.jp/kegg/pathway.html). Selected pathways included: cytokine-cytokine receptor interaction, leukocyte transendothelial migration, Jak-STAT signaling pathways, MAPK signaling pathways, natural killer cell-mediated cytotoxicity, apoptosis, Toll-like receptor signaling pathways, T & B cell receptor signaling pathways, arachidonic acid metabolism, insulin signaling pathways, neuroactive ligand-receptor interactions, focal adhesion, ubiquitin-mediated protein lysis, and chronic myeloid leukemia. By this approach, 68 genes were selected and essentially corresponded to transcription factors, enzymes, regulatory elements, cytokines/chemokines and receptors (Supplementary Table S2).

**Figure 1:**
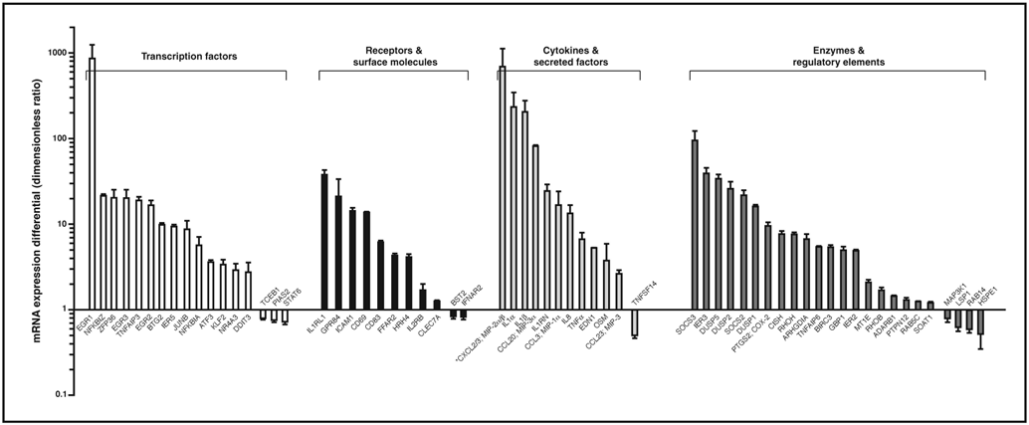
Change in levels of mRNA expression by 64 genes in human neutrophils due to stimulation with inflammatory agonists. Cells were stimulated as described in *Materials and Methods* for 30 min at 37°C. Values are ratios of mRNA levels (stimulated cells/un-stimulated cells) as determined by real-time PCR, averaged±SEM for six independent experiments performed under identical conditions with a different single donor of cells. *Selected MIP-2 primers do not discriminate between the highly homologous alpha and beta isoforms.

We sought to validate the gene chip results using real-time PCR for analysis of the selected genes. This analysis corroborated a significant differential expression between resting and stimulated cells for 64 of the 68 genes. Integrated real-time PCR results are presented in Figure 1. Overall, these results provided strong corroboration of the gene chip assays, in terms of both the identification of differentially expressed genes and the magnitude of the differential expression. The majority of these genes were up-regulated in stimulated cells, with increases reaching 800-fold in some cases. Among these are members of the early growth response family of transcription factors, the IL-1 receptor-like 1, and cytokines/chemokines IL-1α/β, CXCL8 (IL-8), CCL20/23 and CXCL2/3, as well as suppressor of cytokine signaling (SOCS) 3. Other up-regulated genes encode for a number of acute phase proteins such as IER2, 3 and 5, receptors GPR84 and ICAM1, as well as for enzymes such as dual-specificity phosphatases (DUSP) 1, 2 and 5. In comparison, only a small number of genes were down-regulated following cell stimulation and the observed decreases were of relatively modest magnitude. This constitutes, to our knowledge, a first comprehensive gene expression profiling for inflammatory neutrophils.

### Genes modulated by anti-inflammatory agents

**Figure 2:**
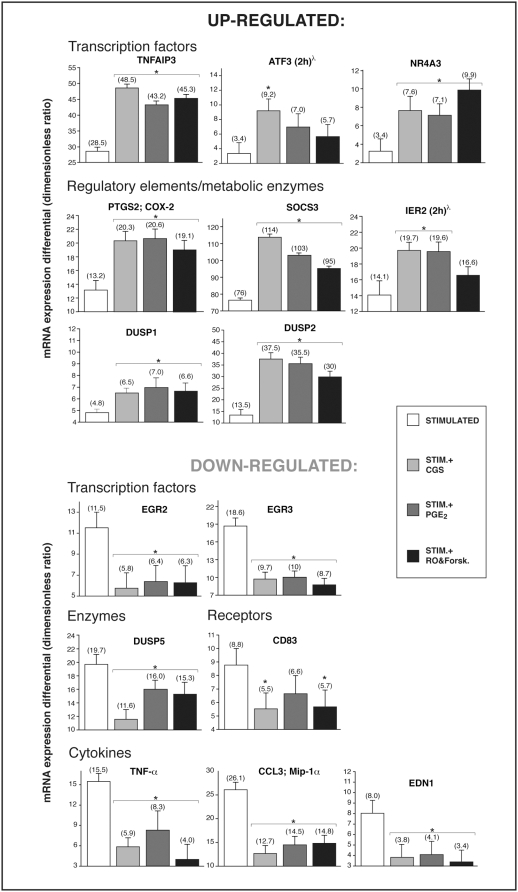
Regulation of genes by anti-inflammatory agents in stimulated human neutrophils. Cells were pretreated with CGS 21680 (1 µM), PGE_2_ (10 µM) or a mixture of 10 µM RO-20-1724 and 50 µM forskolin, then stimulated as described in *Materials and Methods* for 30 min at 37°C, or for 2 h where indicated (^λ^). Top panels show genes that are up-regulated by the anti-inflammatory treatments and bottom ones show genes that are down-regulated.Values are ratios of mRNA levels (treated cells/un-stimulated cells) as determined by real-time PCR, averaged±SEM for six independent experiments performed under identical conditions with a different single donor of cells. *Significantly different from samples stimulated in the absence of any anti-inflammatory agent.

We used the A_2A_R agonist CGS 21680, PGE_2_, or the cAMP-elevating compounds RO 20-1724 (phosphodiesterase IV inhibitor) and forskolin (adenylate cyclase activator), which are potent anti-inflammatory agents known to modulate neutrophil activation [17], in order to determine their impact of the gene expression profile of stimulated neutrophils. Analysis by gene chips revealed that, of the 64 genes differentially expressed in stimulated cells, 28 appeared influenced by at least one of these agents (Supplementary Table S3). Several genes behaved as predicted; the inducible cyclooxygenase COX-2 being up-regulated, while TNF-α and MIP-1α down-regulated by A_2A_R activation, which is in line with earlier findings [21], [23]. These gene chip results were then confronted with real-time PCR experiments performed with new samples from six different donors, which confirmed significant differential expression for 15 of the 28 genes (Figure 2). A_2A_R engagement, PGE_2_ or cAMP-elevating agents each increased mRNA expression of immunomodulatory transcription factors NR4A3, ATF3, TNFAIP3 and IER2, of the enzyme COX-2, of dual-specificity phosphatases 1 and 2 and of the regulatory element SOCS3. Conversely, a number of genes were down-regulated by the anti-inflammatory treatments, notably the pro-inflammatory cytokines, TNF-α, macrophage inflammatory peptide-1α (CCL3/MIP-1α), endothelin-1, members of the early-growth response family of transcription factors (EGR2, EGR3) and the DUSP5 enzyme (Supplementary Table S4). Remarkably, the three distinct anti-inflammatory approaches each had a comparable overall impact on the gene expression profile.

**Figure 3:**
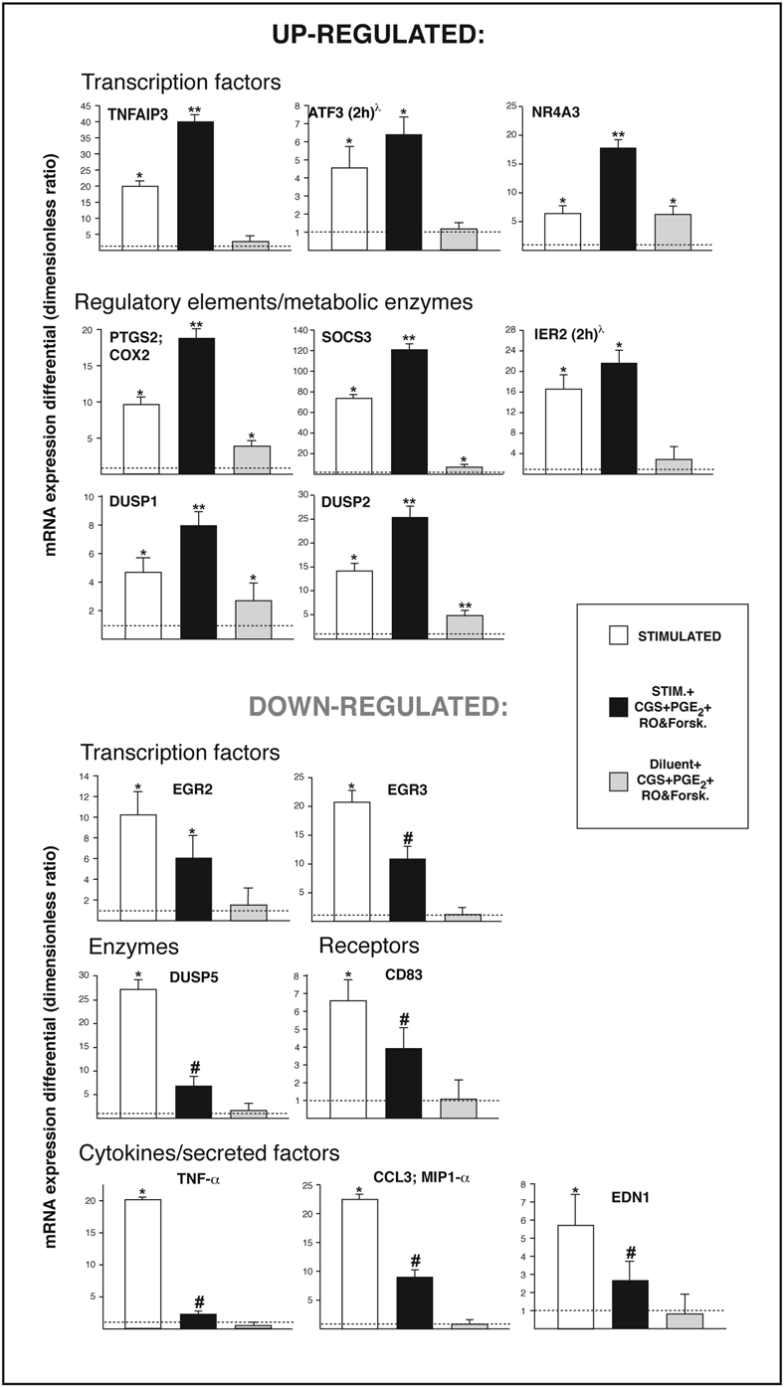
Impact of a combination of anti-inflammatory agents on gene expression in stimulated human neutrophils. Cells were pretreated with CGS 21680 (1 µM), PGE_2_ (10 µM), RO-20-1724 (10 µM) and forskolin (50 µM) simultaneously, then stimulated as described in *Materials and Methods* for 30 min at 37°C, or for 2 h where indicated (^λ^). Top panels show genes that are up-regulated by the anti-inflammatory treatments and bottom ones show genes that are down-regulated. The dotted line indicates the level of expression observed in un-stimulated cells ( = 1). Values are ratios of mRNA levels (treated cells/un-stimulated cells) as determined by real-time PCR, averaged±SEM for three independent experiments performed under identical conditions with a different single donor of cells. *Significantly higher than in un-stimulated samples. **Significantly higher than in un-stimulated samples and in samples stimulated in the absence of anti-inflammatory agent. “#” Significantly higher than in un-stimulated samples but significantly lower than in samples stimulated in the absence of anti-inflammatory agent.

Gene chips and real-time PCR showed similar effects of PGE_2_ or pharmacological elevation of intracellular cAMP on most of the genes affected by A_2A_R engagement, suggesting that even when distinct receptors are engaged, signaling pathways eventually merge and cAMP-dependent processes take part in a central anti-inflammatory response. In order to address this point specifically, we next stimulated neutrophils in the simultaneous presence of all three types of anti-inflammatory agent. Messenger RNA levels of the 15 genes identified earlier were determined by real-time PCR. This experiment produced essentially the same result as obtained with each anti-inflammatory strategy alone (Figure 3), further advocating for an important role of these genes in limiting cell activation. Indeed, no additive or synergistic effect was obtained for the majority of the genes. The exceptions were NR4A3 and DUSP5, for which the simultaneous presence of the anti-inflammatory agents proved more potent than any individual agent. Overall, these results support the concept of a relative redundancy between the distinct anti-inflammatory agents and more specifically their participation in a central and largely cAMP-dependent cellular immunomodulatory response.

**Figure 4:**
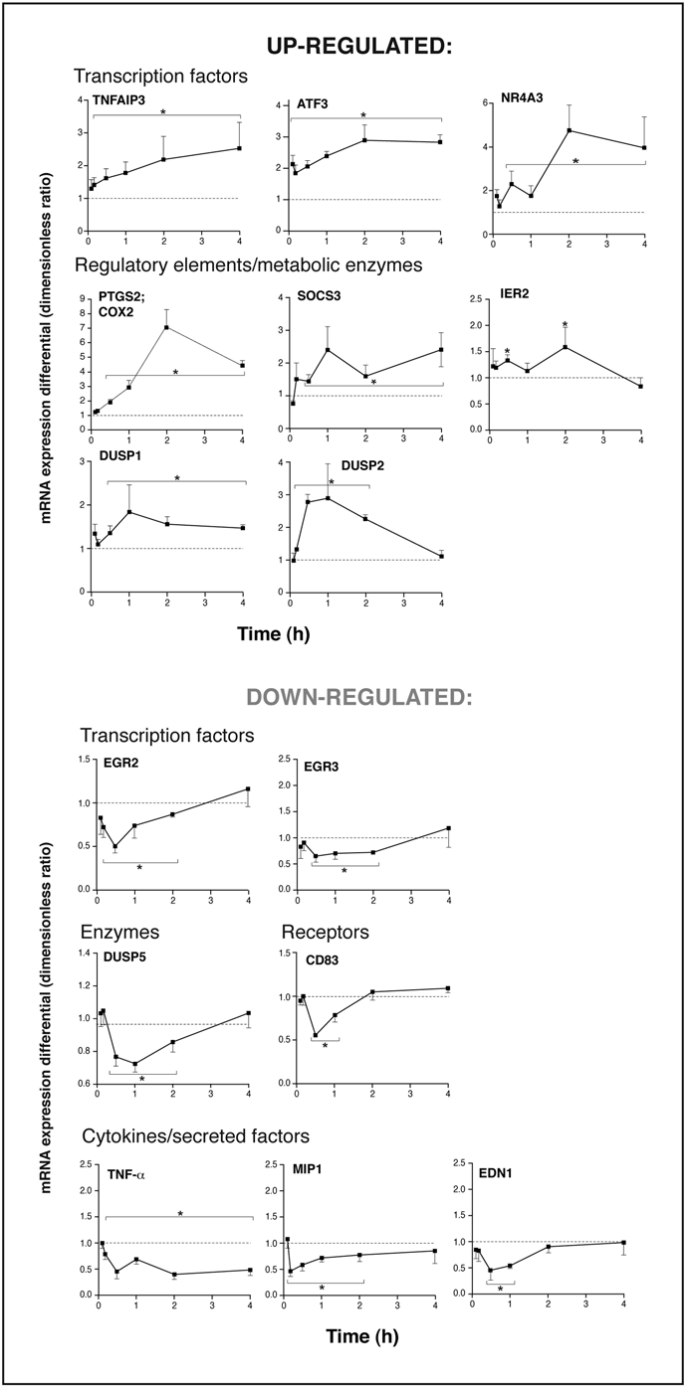
Kinetics of the effect of A_2A_R engagement on gene expression. Cells were pretreated with CGS 21680 (1 µM) then stimulated as described in *Materials and Methods* for 30 minutes at 37°C. Values are time-matched ratios of mRNA levels (CGS21680-treated cells/stimulated cells) as determined by real-time PCR, averaged±SEM for three independent experiments performed under identical conditions with a different single donor of cells. *Significantly different from cells stimulated in the absence of CGS 21680.

Time-course experiments were undertaken in which cells were stimulated for periods of time ranging from 5 min to 4 h, alone or in presence of the A_2A_R agonist CGS 21680. Messenger RNA levels for genes of interest were measured by real-time PCR and samples stimulated in the absence or presence of CGS 21680 were compared in a time-matched manner. Depending on the gene, A_2A_R activation elicited transient (<2 h) or sustained (≥4 h) responses, indicative of gene-specific regulatory processes (Figure 4). However, the impact on gene expression was typically rapid, in most cases becoming apparent in less than 30 minutes.

## Discussion

The scope of this work encompasses the development of novel therapeutic strategies based on enabling endogenous anti-inflammatory pathways in the treatment of inflammatory conditions such as rheumatoid arthritis, in which unchecked activation of cells can cause significant tissue damage. By profiling gene expression in stimulated neutrophils, we delineated a group of genes that respond to immunomodulatory signals. Gene identification was achieved using a gene chip approach and was corroborated by real-time PCR. In response to three distinct anti-inflammatory approaches, stimulated neutrophils shifted their expression of specific genes. Cells responded to these different anti-inflammatory signals in a strikingly similar fashion, which suggests the engagement of a central endogenous system responsible for uncoupling selected neutrophil inflammatory functions.

The pro-inflammatory, receptor-activating agonists *Escherichia coli* lipopolysaccharide, granulocyte-macrophage colony-stimulating factor, tumor-necrosis factor α, formyl-methionyl-leucyl-phenylalanine and interleukin 1β were chosen for their importance in inflammatory processes and for their well-documented stimulatory effects on neutrophils [21]–[23], [35]–[37]. In the course of an inflammatory response, it is assumed that recruited neutrophils are more likely to encounter a multitude of extracellular messengers, rather than a single one. Even so, most studies of anti-inflammatory agents have used a single agonist and for that matter, a single anti-inflammatory agent, thereby locking down data interpretation on pathways solicited by that particular agonist only. In this study, we elected to stimulate neutrophils concurrently with a group of agonists chosen for their well-described ability to engage distinct classes of receptors in neutrophils. GM-CSF interacts with a receptor comprising tyrosine kinase activity [38], fMLP signals through seven-transmembrane domain receptors FPR1 and FPRL1 linked to heterotrimeric GTP-binding proteins [39], LPS associates with LPS-binding proteins, then with the Toll-Like receptor 4 and CD14 molecule [40], TNF-α binds to its ceramide-linked receptors TNFRSF1A/TNFR1 and TNFRSF1B/TNFBR [41] and finally, IL-1β engages its own family of receptors (IL-1R1, IL-1R2, IL1-RL1) in the immunoglobulin domain superfamily [42]. This multilateral stimulation, likely closer to what inflammatory cells face, favorably elicits a robust and more comprehensive cellular involvement, a suitable situation for the study of anti-inflammatory processes.

The impact of the anti-inflammatory agents on cell response was clearly multi-pronged, even considering only their effects on gene expression. Genes involved encode transcription factors, enzymes and regulatory factors, receptors and cytokines. It is of interest that some of the genes were actually up-regulated by the anti-inflammatory agents, implying an active cellular reaction rather than a mere response to inhibition. Such up-regulated genes included pivotal transcription factors that have been reported to modulate numerous cell functions. For example, TNFAIP3 displays potent anti-inflammatory properties in a number of different cell types, through the inhibition of NF-kB activation and prevention of TLR-mediated responses [43]–[48]. ATF3 plays a protective role in ischemia-reperfusion injury and in response to stress [49], [50]. NR4A3 prevents NF-kB activation, thereby reducing inflammatory responses such as the generation of pro-inflammatory cytokines. Available evidence also suggests a protective role for this family of transcription factors in atherogenesis [51], [52]. A number of enzymes and regulatory elements were also up-regulated by the anti-inflammatory agents. The inducible cyclooxygenase isoform COX-2 is a pivotal and rate-limiting enzyme in the inflammation-related generation of PGE_2_, the latter having potent anti-inflammatory activities in leukocytes and other inflammatory cells [21], [22], [35], [53]. Suppressor of cytokine signaling 3 is the main isoform of that family of regulatory factors expressed in neutrophils and functions by inhibiting JAK2 kinase activity [54]–[57]. DUSP 1 and 2 can inactivate mitogen-activated protein kinases by dephosphorylating phosphothreonine and phosphotyrosine residues. Phosphatases of the DUSP family display specificity for different MAP kinases and differ in tissue and sub-cellular distributions, although DUSP 2 is expressed predominantly in hematopoietic tissues [58], [59]. Finally, IER2 attenuates the signaling activity of G proteins by binding to GTP-bound G alpha subunits and by increasing the rate of conversion from GTP to GDP [60]. Clearly, the up-regulation of gene expression by adenosine and PGE_2_ occurs in a way that can alter cellular programming at several levels.

Inhibition of key inflammatory factors is also likely pivotal for mediating the potent anti-inflammatory activities of adenosine and PGE_2_ in neutrophils. Indeed, activities of early-growth-response transcription factors 2 and 3 are believed to have positive involvement in differentiation, mitogenesis and angiogenesis [61], while the phosphatase DUSP5 has been linked positively to immunity through T cell development [62]. In the present study, the only receptor to respond to anti-inflammatory agents (by down-regulation of mRNA) was CD83, which is considered a marker of mature dendritic cells and thought to be involved in the regulation of T- and B-lymphocyte maturation. Although its expression on neutrophils is known, its role is currently unknown [63], [64]. As we reported earlier, TNF-α expression is diminished by adenosine and may well be one of the key targets of this autacoid. Indeed, TNF-α is a pivotal pro-inflammatory cytokine involved in a number of inflammatory and autoimmune diseases, diabetes and cancer [65], [66]. Similarly, MIP-1α is another important cytokine involved in the acute inflammatory state and in the recruitment and activation of neutrophils [67]–[69]. Endothelin 1 is a potent vasoconstrictor that has been linked to graft rejection and to inflammatory events including pain, fever, cell migration and rheumatoid arthritis. It stimulates several mechanisms on neutrophils, including adhesion and migration [70]–[74].

Genes that were found to be affected by A_2A_R engagement, PGE_2_ or pharmacological elevation of the intracellular cyclic AMP concentration together provide a first picture of the overall impact these signals have on gene expression. Furthermore, single anti-inflammatory signals affected the expression of the same group of genes, supporting the hypothesis that these genes play a role in the coordination of a cellular response, this role being to limit cell activation. It is therefore possible that the expression profile observed in neutrophils will find similarities in other cell types and tissues, and engage a resolution response. The current picture is still partial; indeed, a number of affected sequences either code for proteins not yet characterized or are altogether not translated. Also, further studies will be necessary to confirm their involvement in resolving inflammation. Nonetheless, genes identified in the present study are likely to provide a better understanding of anti-inflammatory signaling.

In summary, we have identified a series of genes for which expression is altered by major anti-inflammatory signals. All of theses signals affected the gene expression profile in remarkably similar fashion. Characterization of these signaling pathways will improve our understanding of the capacity of tissues to terminate inflammation and may lead to the identification of better therapeutic targets for the treatment of inflammatory diseases associated with unrepressed neutrophil activation.

## Materials and Methods

### Materials

#### Anti-inflammatory agents

Compound CGS 21680 (2-[p-(2-carboxyethyl) phenethylamino]-5′-N-ethyl carboxamidoadenosine) was from Research Biochemicals International (Natick, MA, USA). Prostaglandin E_2_ (PGE_2_) was purchased from Cayman Chemicals (Ann Arbor, MI, USA). Forskolin and RO 20-1724 were obtained from EMD Chemicals (San Diego, CA, USA).

#### Inflammatory agonists

Lipopolysaccharide (LPS) from *Escherichia coli* O111:B4 and formyl-methionyl-leucyl phenylalanine (fMLP) were obtained from Sigma-Aldrich (Oakville, ON, Canada). Recombinant human granulocyte-macrophage colony-stimulating factor (GM-CSF), tumor necrosis factor α (TNF-α) and interleukin 1β (IL-1β) were purchased from PeproTech (Rocky Hill, NJ, USA).

Adenosine deaminase was purchased from Roche Applied Science (Laval, QC, Canada).

### Neutrophil isolation

Polymorphonuclear leukocytes were isolated as originally described [75] with modifications [22]. Informed consent was obtained in writing and all experiments involving human tissues were approved by the Laval University Ethics Committee. Data collection and analyses were performed anonymously. Briefly, venous blood from healthy volunteers, collected on isocitrate anticoagulant solution was centrifuged (250×*g*, 10 min) and the resulting platelet-rich plasma was discarded. Leukocytes were obtained following erythrocyte sedimentation in 2% Dextran T-500 (Sigma-Aldrich). Granulocytes were then separated from other leukocytes by centrifugation on a 10 ml cushion of lymphocyte separation medium (Wisent, St-Bruno, QC, Canada). Contaminating erythrocytes were removed by 15 seconds of hypotonic lysis. Purified granulocytes (>95% neutrophils, <5% eosinophils) contained less than 0.1% monocytes, as determined by esterase staining. Viability was greater than 98%, as determined by tryptan blue dye exclusion. The whole cell isolation procedure was carried out at room temperature under sterile conditions.

### Cell stimulations

Neutrophils were re-suspended at a concentration of 30×10^6^ cells/ml in Hank's balanced salt solution at 37°C, containing 1% fetal bovine serum, 10 mM HEPES pH 7.4, 1.6 mM Ca^2+^ and no Mg^2+^. Adenosine deaminase (0.1 U/ml) was added to cell suspensions 20 min prior to stimulation in order to prevent accumulation of extracellular adenosine in cell suspensions, thus minimizing the modulating effects of adenosine on neutrophil activities [76]. Anti-inflammatory compounds dissolved in dimethylsulfoxide were added to cell suspensions 10 min before stimulation with a mixture of LPS (100 ng/ml), GM-CSF (1.4 nM), TNF-α (100 ng/ml), fMLP (100 nM) and IL-1β (30 nM). Organic solvent concentration was identical in all samples and did not exceed 0.1% (v/v). Stimulations were for 30 min at 37°C, unless indicated otherwise.

### RNA isolation

Following stimulation, neutrophil total RNA was isolated using Trizol (Invitrogen, Burlington, ON, Canada) according to the manufacturer's protocol, with modifications [22]. Briefly, a pellet containing 30×10^6^ neutrophils was homogenized in 1 ml Trizol and 200 µl of chloroform were added. After mixing, the sample was centrifuged at 12,000×*g* for 15 min (4°C) and the upper aqueous phase (450 µl) was transferred to a tube containing an equal volume of isopropanol, mixed thoroughly using a vortex device and centrifuged at 12,000×*g* for 10 min (4°C). The supernatant was discarded and the precipitated RNA pellet was washed twice using 500 µl of 75% ethanol and centrifuged at 12,000×*g* for 5 min (4°C). The final pellet was allowed to air-dry for 5–10 min and was then re-suspended in RNAse-free water. RNA was quantitated using a Qubit™ Fluorometer (Invitrogen).

### DNA microarrays

Equal quantities of total RNA obtained from neutrophils of five donors were pooled together and purified on QIAGEN RNeasy column (QIAGEN, Mississauga, ON, Canada). Ten ng of total RNA were converted to cDNA using Superscripts reverse transcriptase (Invitrogen) and T7-oligo-d(T)_24_ primers (Applied Biosystems, Austin, TX, USA). Second-strand synthesis was performed using T4 DNA polymerase and *E. coli* DNA ligase and then blunt-ended by T4 polynucleotide kinase. cDNA was purified by phenol-chloroform extraction using phase lock gels (Brinkmann, Westbury, NY, USA), then transcribed in vitro for 16 h at 37°C by using the IVT Labelling Kit (Affymetrix, Santa Clara, CA, USA) to produce biotinylated cRNA. Biotin-labelled cRNA was isolated using the RNeasy Mini Kit column (QIAGEN). Purified cRNA was fragmented to 30–200 nucleotide lengths using a fragmentation buffer. The quality of total RNA, cDNA synthesis, cRNA amplification and cRNA fragmentation was monitored and confirmed by capillary electrophoresis (Bioanalyzer 2100, Agilent Technologies, Santa Clara, CA, USA). Fifteen µg of fragmented cRNA were hybridized for 16 h at 45°C with constant rotation on a Human Genome U133 Plus 2.0 GeneChip Array (Affymetrix). After hybridization, gene chips were processed with the Affymetrix GeneChip Fluidic Station 450 (protocol EukGE-WS2v5_450). Briefly, staining was made with streptavidin-conjugated phycoerythrin (SAPE, Invitrogen) followed by amplification with a biotinylated anti-streptavidin antibody (Vector Laboratories, Burlingame, CA, USA) and by a second round of SAPE. Chips were scanned using a GeneChip Scanner 3000 G7 (Affymetrix) enabled for High-Resolution Scanning. Images were extracted with the GeneChip Operating Software (Affymetrix GCOS v1.4). Quality control of microarray chips was performed using the AffyQCReport software [77]. All Microarray data is conform to the MIAME guidelines; unsupervised, raw data was deposited in the GEO database (geo@ncbi.nlm.nih.gov), submission number: GSE14465.

### Interpretation of microarray results

Sequences with a level of expression of 200 and over were considered to be positively expressed. A sequence was considered differentially expressed when the ratio of expression level between experimental conditions was ≥2 (≥two-fold increase) or ≤0.5 (two-fold decrease). Gene identification and expression levels were analyzed using the Gene Set Analysis Toolkit, developed and maintained by members of the Department of Biomedical Informatics and the Department of Biostatistics of the Vanderbilt University Medical Center (http://bioinfo.vanderbilt.edu/webgestalt) [34].

### Real-Time PCR

First-strand cDNA synthesis was performed using 1 µg of total RNA with Superscript II (Invitrogen) following the manufacturer's instructions, using 500 ng of random hexamers. Real-time PCR was performed as described elsewhere [78]. Briefly, cDNA amplification was carried out in a Rotor-Gene 3000 operated with Rotor-Gene software version 6.0.19 (Corbett Research, Mortlake, NSW Australia) using 35 cycles of 95°C, 58°C and 72°C for 20 seconds each. Each sample consisted of 40 ng of cDNA, 2 µl of 10× buffer (100 mM Tris, 500 mM KCl, 30 mM MgCl_2_, 1.5% Triton X-100), 0.5 mM dNTP, 500 nM of primers, 0.1 unit of r*Taq* DNA polymerase (GE Healthcare, Piscataway, NJ, USA) and SYBR Green I dye (Invitrogen; 1∶30,000 dilution) in a reaction volume of 20 µL. Reaction specificity was ascertained by performing the Melt® procedure (58–99°C, 1°C/5 s) at the end of the amplification protocol, according to the manufacturer's instructions. For each gene of interest, specific primers were designed as described previously [78]. Briefly, primers were selected systematically within the coding region, with a theoretical melting point of 58°C, GC content of 50% (±10%) and length of 18–24 bp, for an average product length of 200 bp. Primers thus designed were all tested with gradient PCR prior to their use in real-time PCR and are listed in Supplementary Table S5.

### Experimental design

The following experimental treatment conditions were examined: Unstimulated control cells; stimulated cells; cells stimulated in the presence of either CGS 21680, PGE_2_, or forskolin & RO 20-1724; cells stimulated in the simultaneous presence of CGS 21680, PGE_2_ and forskolin & RO 20-1724; unstimulated cells in the simultaneous presence of CGS 21680, PGE_2_ and forskolin & RO 20-1724.

DNA microarray experiments were repeated twice, each using RNA pooled from neutrophils of five donors. Real-time PCR experiments were repeated six times for cell stimulation in the absence of anti-inflammatory agents and for stimulation in the presence of CGS 21680, PGE_2_, or forskolin plus RO 20-1724 (each repetition with neutrophils from one of six donors) and repeated three times for the time-course study of CGS 21680 (each repetition with neutrophils from one of three donors),

### Statistical analysis

Where applicable, statistical analysis was performed using the Student's non-paired t-test (two-tailed) and differences were considered significant (marked by an asterisk) when *p*<0.05.

## Supporting Information

Table S1 Gene-chip-based identification of differentially regulated genes in stimulated human neutrophils. Genes are listed in decreasing order of the mRNA expression ratio (stimulated/un-stimulated cells). Cells were stimulated as described in Materials and Methods for 30 min at 37 degrees C. RNA from five donors was pooled for analysis. Results are from one experiment.
(0.09 MB XLS)

Table S2 Inflammatory genes that are differentially- expressed in stimulated human neutrophils (Genechip results). Cells were stimulated as described in Materials and Methods for 30 min at 37 degree C. RNA from five donors was pooled for analysis. Genes from Supplementary Table S1 were selected for their potential involvement in inflammatory processes, and based on differential regulation in stimulated human neutrophils, as revealed by genechip analysis. Genes are listed in decreasing order of the mRNA expression ratio (stimulated/un-stimulated cells). Results are from one experiment.
(0.15 MB XLS)

Table S3 Differentially-expressed genes in stimulated human neutrophils treated with anti-inflammatory agents (Genechip results) Cells were pretreated with CGS 21680 (1 uM), PGE2 (10 uM) or a mixture of 10 uM RO-20-1724 and 50 uM forskolin, then stimulated for 30 min with the inflammatory cocktail for 30 min at 37 degree C, as described in Materials and Methods. RNA from five donors was pooled for analysis. Genes are listed in decreasing order of the mRNA expression ratio (stimulated/un-stimulated cells). Results are from one experiment.
(0.16 MB XLS)

Table S4 List of genes, their associated proteins and protein functions, differentially regulated in stimulated human neutrophils and influenced by anti-inflammatory agents, as confirmed by real-time PCR.
(0.03 MB XLS)

Table S5 List of PCR primers.
(0.16 MB XLS)
